# A case of asymptomatic giant renal angiomyolipoma with extrusion of fat content with literature review

**DOI:** 10.1093/jscr/rjae732

**Published:** 2024-11-26

**Authors:** Manzoor Ahmad, Kartik Varshney, Shagufta Qadri, Wasif Mohammad Ali, Imad Ali

**Affiliations:** Department of Surgery, Jawaharlal Nehru Medical College, Medical College Road, Aligarh Muslim University, Aligarh 202002, India; Department of Surgery, Jawaharlal Nehru Medical College, Medical College Road, Aligarh Muslim University, Aligarh 202002, India; Department of Pathology, Jawaharlal Nehru Medical College, Medical College Road, Aligarh Muslim University, Aligarh 202002, India; Department of Surgery, Jawaharlal Nehru Medical College, Medical College Road, Aligarh Muslim University, Aligarh 202002, India; Department of Surgery, Jawaharlal Nehru Medical College, Medical College Road, Aligarh Muslim University, Aligarh 202002, India

**Keywords:** giant angiomyolipoma, nephrectomy, renal artery embolization, nephron-sparing surgery

## Abstract

Angiomyolipoma is a benign mesenchymal tumour of kidney that consists of adipose tissue, muscle cells and blood vessels. Renal angiomyolipomas represent almost one percent of all renal tumours. We reported a case of a 50-year-old woman complaining of mild abdominal discomfort with no other symptoms and no remarkable medical history. Clinical examination was inconclusive and no lump was palpable in abdomen. Ultrasonography raised the suspicion of existence of a large, homogeneous, hyperechoic tissue mass arising from right kidney. Abdominal computed tomography scan suggested the presence of giant angiomyolipoma of right kidney. The histopathological examination confirmed the diagnosis of renal angiomyolipoma. The patient underwent open simple nephrectomy to remove the tumour without any complications. We documented a rare case of retroperitoneal angiomyolipoma with extrusion of fat content from the renal hilum breaching the cortex as peculiarity which presented just as mild abdominal discomfort.

## Introduction

Angiomyolipoma are benign renal neoplasms made up of a heterogeneous group of abnormal blood vessels, smooth muscle, and adipose tissue. While the majority of patients show no symptoms, certain tumours might cause acute abdominal pain or hypotension because of significant intra-lesional bleeding or retroperitoneal invasion. Tumour size has a major impact on how patients with renal angiomyolipoma are treated. Less than 4 cm tumours are usually asymptomatic and can be monitored closely, while larger tumours may necessitate the use of one or more of the available treatment modalities, like partial nephrectomy, trans-arterial embolization, or radiofrequency ablation [1].

These tumours are mostly benign and rare in variety. Approximately 80% of these tumours are sporadic in nature. In certain instances, however, they have been associated with tuberous sclerosis complex and they primarily affect females [2]. According to the previous researches, the maximum rate of growth of renal angiomyolipoma (AML) is 4 cm per year [1, 3]. When the tumour size exceeds 10 cm, renal AMLs are referred to as ‘giant’ AMLs. A very few giant renal AMLs have been documented in the literature so far [4].

## Case report

A 50-year-old woman with no comorbidity arrived at our hospital’s outpatient department complaining of mild abdominal discomfort. On clinical examination, there was no tenderness or palpable lump noted in abdomen. She came with an ultrasonographic scan suggestive of the presence of an echogenic mass of 130 × 95 mm with a broad, well-defined heterogeneous echo texture, along with several hypoechoic regions in mid cortex of right kidney.

**Figure 1:**
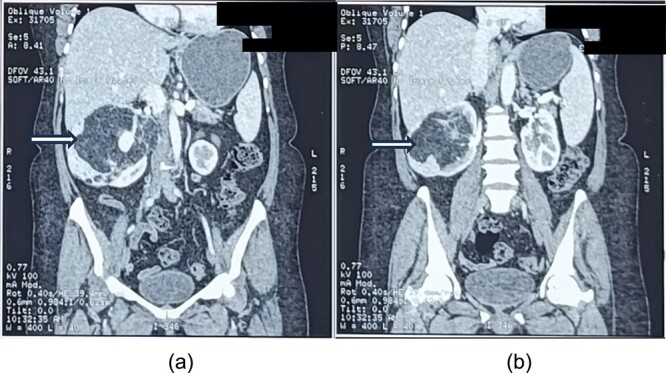
a and b showing abdominal CT scan cuts, showing mixed density mass arising from the right kidney (white arrows).

On contrast-enhanced computed tomography (CECT) of the abdomen, there was a large (size ~9.0 × 13.7 × 8.2 cm, APXTRXCC), well defined heterogeneous predominantly hypodense lesions with a large fat component with CT value of fat (−45 to −60) and an enhancing solid part with tortuous vessels at the periphery of lesion arising from the interpolar region of right kidney causing architectural distortion of most of the renal parenchyma (Fig. 1a and b), diagnosed as AML.

The lesion was causing mass effect on the inferior vena cava posteriorly, 1st and 2nd part of duodenum, head of the pancreas displacing them anteriorly and right renal vein anteromedially. There were no clinic-radiological features of tuberous sclerosis complex (TSC).

Her haemoglobin level was 13.0 gm/dl and other investigations like renal function tests, urine routine and microscopy were within normal limits.

We took the decision for open simple nephrectomy which was influenced by large fat content and complexity of the tumour, making the nephron-sparing surgery, trans-arterial embolization and radiofrequency ablation impractical. Laparoscopic approach was not considered because of proximity of fat dominant tumour to critical structures like inferior vena cava, duodenum and pancreas.

Intraoperatively, after mobilizing the right side colon, a large renal mass was seen with fat extrusion from the renal capsule. The mass was covering the inferior vena cava behind and displacing duodenum and head of pancreas anteriorly. This extrusion is not commonly reported in AML cases and adds a novel element to the literature on AML pathophysiology. The right kidney containing the fatty tumour was resected en-mass, and haemostasis was secured. The estimated blood loss was <100 ml and post-operatively, the patient’s recovery was uneventful. The patient was discharged on the fourth day under satisfactory condition.

**Figure 2:**
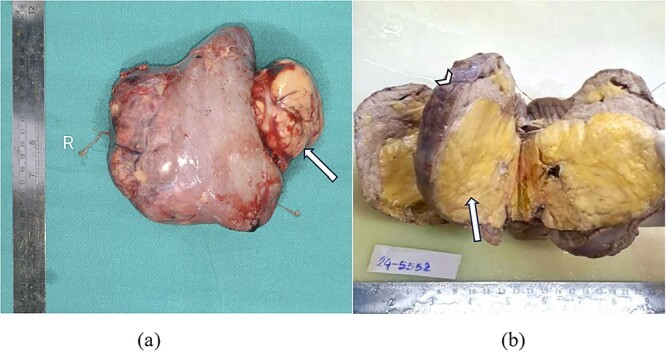
(a) Gross resected nephrectomy specimen showing tumour fat extrusion breaching renal hilum (arrow). (b) Cut section of nephrectomy specimen showing yellow to tan tumour areas (arrow) replacing entire renal parenchyma (arrowhead).

Resected nephrectomy specimen showed surface nodularity, covered by renal capsule and showed focal extrusion of fat content outside renal tissue towards hilum breaching renal capsule as shown in Fig. 2a.

Cut section of nephrectomy specimen showed almost whole of renal parenchyma replaced by yellow to tan tumour area, leaving behind only a thin rim of normal appearing cortex at periphery as shown in Fig. 2b.

**Figure 3:**
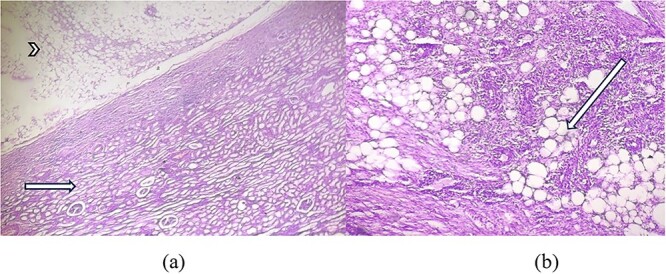
(a) H&E × 40 section shows tumour area (arrowhead) consisting of adipose tissue with compressed glomeruli and tubules (short arrow). (b) H&E × 100 section shows mature adipose tissue (long arrow) along with haphazardly arranged smooth muscle fibres and infiltrated by dense inflammatory cells.

As shown in Fig. 3a, microscopic examination of H&E stained section showed tumour area consisting of adipose tissue and spindle cells with compressed renal parenchyma containing glomeruli and tubules. Another H&E stained section showed areas with mature fatty adipose tissue along with haphazardly arranged smooth muscle fibres having cigar shaped nuclei and fibrillary eosinophilic cytoplasm and many small vessels, infiltrated by dense mixed inflammatory cells consisting of lymphocytes and few plasma cellsas shown in Fig. 3b.

## Discussion

Renal AML is a non-malignant neoplasm derived from mesenchymal elements that was first reported in 1951 [5]. AML is also referred to as a ‘hamartoma’ because of its heterogeneous composition, which includes blood vessels, smooth muscle, and adipose tissue [6]. Typically, these tumours are classified as either sporadic or in the context of TSC. Our case under discussion falls into the former group.

This large tumour has blood vessels with thin walls, making it prone to vascular rupture and bleeding. The formation of haemorrhagic aneurysms concomitant with the expanding AML increases the risk of compression symptoms and haemorrhage and may require prophylactic or therapeutic trans-arterial embolization [7, 8]. Haematuria, shock, and back pain are the three clinical signs that are most important in suggesting retroperitoneal haemorrhage, also known as Wunderlich’s syndrome. Patients with renal AML typically seek treatment for these clinical signs.

There are only a few documented cases of giant renal AMLs in the literature [4]. Table 1 presents a comparative description of available case reports found in the literature in last 10 years.

**Table 1:** Tabular column displaying similar instances of large angiomyolipoma in literature

Case report	Size	Clinical presentation	Procedure done
Alshehri *et al.* 2020 [9]	29 × 23 × 21 cm	large right abdominal swelling and vague abdominal pain for past 3 years	Open radical nephrectomy
Chen *et al.* 2017 [10]	29 × 20 × 10 cm	Bloating sensation	Open simple nephrectomy
Kori *et al.* 2015 [11]	23 × 20 × 12 cm	Dull pain and palpable lump in left side of the abdomen	Open radical nephrectomy
Taneja *et al.* 2013 [12]	39 × 25 × 9 cm	bloating sensation in the abdomen and a steadily increasing abdominal girth for about 3 years	Open simple nephrectomy

Tumours of this size are often symptomatic, presenting with flank pain, haematuria, or palpable masses, and are more likely to rupture, causing life-threatening haemorrhage. The present case is unusual due to the absence of such symptoms despite the tumour’s size and mass effect on surrounding structures. This discrepancy emphasizes the variability in clinical presentation among patients with giant AMLs. The echogenicity of the mass on ultrasound and its CT scan density resembling that of fat are indicative findings.

None of the reported cases documented the extrusion of fat content outside the renal capsule as seen in our case. This unusual presentation emphasizes the importance of comprehensive imaging and further histopathological evaluation to guide treatment. The management of giant AMLs should be individualized based on tumour size, location, patient symptoms, and risk factors such as haemorrhage. There was no evidence of tumour recurrence at 6-month post-operative follow-up.

## Conclusion

This case adds to the limited literature on giant renal AMLs, particularly those with unique characteristics such as fat extrusion through the renal cortex with no significant symptoms. Surgical resection remains the treatment of choice for large, complex AMLs, especially when they pose a risk of rupture or compress vital structures.

## References

[1] Seyam RM , Bissada NK, Kattan SA, et al. Changing trends in presentation, diagnosis and management of renal angiomyolipoma: comparison of sporadic and tuberous sclerosis complex associated forms. Urology 2008;72:1077–82. 10.1016/j.urology.2008.07.049. 18805573

[2] Çalışkan S , Gümrükçü G, Özsoy E, et al. Renal angiomyolipoma. Rev Assoc Med Bras (1992) 2019;65:977–81. 10.1590/1806-9282.65.7.977. 31389508

[3] Ewalt DH , Sheffield E, Sparagana SP, et al. Renal lesion growth in children with tuberous sclerosis complex. J Urol 1998;160:141–5. 10.1016/S0022-5347(01)63072-6. 9628635

[4] Chronopoulos PN , Kaisidis GN, Vaiopoulos CK, et al. Spontaneous rupture of a giant renal angiomyolipoma —Wunderlich's syndrome: report of a case. Int J Surg Case Rep 2016;19:140–3. 10.1016/j.ijscr.2015.12.017. 26764888 PMC4756198

[5] Morgan G , Straumfjord JV, Hall EJ. Angiomyolipoma of the kidney. J Urol 1951;65:525–7. 10.1016/S0022-5347(17)68515-X. 14825528

[6] Kulkarni B , Desai SB, Dave B, et al. Renal angiomyolipomas—a study of 18 cases. Indian J Pathol Microbiol 2005;48:459–63. 16366094

[7] Luca D , Rossetti R, Terrone C. Management of renal angiomyolipoma: a report of 53 cases. BJU Int 1999;83:215–8. 10.1046/j.1464-410x.1999.00932.x. 10233482

[8] Bora A , Soni A, Sainani N, et al. Emergency embolization of a bleeding renal angiomyolipoma using polyvinyl alcohol particles. Diagn Interv Radiol 2007;13:213–6. 18092296

[9] Alshehri M , Hakami B, Aljameel N, et al. Sporadic giant renal angiomyolipoma: a case report and literature review of clinical presentation, diagnosis, and treatment options. Urol Ann 2020;12:167–71. 10.4103/UA.UA_26_19. 32565656 PMC7292434

[10] Chen P , Jin L, Yang Y, et al. Giant renal angiomyolipoma: a case report. Mol Clin Oncol 2017;7:298–300. 10.3892/mco.2017.1305. 28781806 PMC5532701

[11] Kori C , Akhtar N, Vamsidhar PN, et al. Giant exophytic renal angiomyolipoma mimicking as retroperitoneal sarcoma; a case report with review of literature. J Clin Diagn Res 2015;9:1–2. 10.7860/JCDR/2015/11514.5798 PMC4437147 26023631

[12] Taneja R , Singh DV. Giant renal angiomyolipoma: unusual cause of huge abdominal mass. J Clin Imag Sci 2013;3:56. 10.4103/2156-7514.122326. PMC3883275 24404415

